# The Effect of Polyphenol-Rich Interventions on Cardiovascular Risk Factors in Haemodialysis: A Systematic Review and Meta-Analysis

**DOI:** 10.3390/nu9121345

**Published:** 2017-12-11

**Authors:** Wolfgang Marx, Jaimon Kelly, Skye Marshall, Stacey Nakos, Katrina Campbell, Catherine Itsiopoulos

**Affiliations:** 1 School of Allied Health, La Trobe University, Bundoora, VIC 3086, Australia; 17987619@students.latrobe.edu.au (S.N.); c.itsiopoulos@latrobe.edu.au (C.I.); 2 Faculty of Health Sciences and Medicine, Bond University, Robina, QLD 4226, Australia; jkelly@bond.edu.au (J.K.); smarshal@bond.edu.au (S.M.); kcampbel@bond.edu.au (K.C.)

**Keywords:** polyphenol, dialysis, cardiovascular, review, meta-analysis, oxidative stress, inflammation, blood pressure, pomegranate, cocoa, soy, turmeric

## Abstract

End-stage kidney disease is a strong risk factor for cardiovascular-specific mortality. Polyphenol-rich interventions may attenuate cardiovascular disease risk factors; however, this has not been systematically evaluated in the hemodialysis population. Using the Preferred Reporting Items for Systematic Reviews and Meta-Analyses (PRISMA) guidelines, the following databases were searched: Cochrane Library (http://www.cochranelibrary.com/), MEDLINE (https://health.ebsco.com/products/medline-with-full-text), Embase (https://www.elsevier.com/solutions/embase-biomedical-research), and CINAHL (https://www.ebscohost.com/nursing/products/cinahl-databases/cinahl-complete). Meta-analyses were conducted for measures of lipid profile, inflammation, oxidative stress, and blood pressure. Risk of bias was assessed using the Cochrane Collaboration Risk of Bias tool and quality of the body of evidence was assessed by the Grading of Recommendations, Assessment, Development and Evaluation (GRADE) methodology. Twelve studies were included for review. Polyphenol-rich interventions included soy, cocoa, pomegranate, grape, and turmeric. Polyphenol-rich interventions significantly improved diastolic blood pressure (Mean Difference (MD) −5.62 mmHg (95% Confidence Interval (CI) −8.47, −2.78); *I*^2^ = 2%; *p* = 0.0001), triglyceride levels (MD −26.52 mg/dL (95% CI −47.22, −5.83); *I*^2^ = 57%; *p* = 0.01), and myeloperoxidase (MD −90.10 (95% CI −135.84, −44.36); *I*^2^ = 0%; *p* = 0.0001). Included studies generally had low or unclear risks of bias. The results of this review provide preliminary support for the use of polyphenol-rich interventions for improving cardiovascular risk markers in haemodialysis patients. Due to the limited number of studies for individual polyphenol interventions, further studies are required to provide recommendations regarding individual polyphenol intervention and dose.

## 1. Introduction

End-stage kidney disease (ESKD) is a major health burden worldwide, with over 2 million people estimated to be receiving renal replacement therapy [1]. Among those with ESKD, cardiovascular disease (CVD) accounts for almost 50% of all deaths, most commonly sudden cardiac death [2]. Many factors are known to influence the elevated CVD risks in ESKD, including high blood pressure, dyslipidaemia and high levels of oxidative stress [3,4]. The uraemic state, which causes increased production of pro-inflammatory cytokines and promotes oxidative stress, may trigger the onset and progression of atherosclerosis and CVD [5]. While adequate dialysis therapy ameliorates the accumulation of uremic toxin and pro-inflammatory cytokines, the dialysis process itself can induce a chronic state of inflammation [6]. This can be further compromised by the loss of key antioxidants during haemodialysis [7], which further exacerbates inflammation and therefore, increases the risk of CVD in dialysis patients. 

Lifestyle modification, including adherence to a cardio-protective diet may provide potential improvements in CVD risk factors in dialysed ESKD patients [8]. However, common limitations to developing nutrition management plans in dialysis, particularly haemodialysis, arise when attempting to implement a cardio-protective diet [9]. Many nutrient restrictions placed on haemodialysis patients have the knock-on effect of limiting antioxidant vitamins (e.g., ascorbic acid, tocopherols), minerals (e.g., selenium), and various non-nutritive polyphenols, which may be attributable to the commonly higher levels of potassium in nutrient-rich fruit and vegetables [10]. Therefore, a low risk dietary intervention which may improve intake of potentially cardioprotective compounds may improve CVD outcomes in the haemodialysis patient. 

A healthy dietary pattern, such as the Mediterranean and Dietary Approaches to Stop Hypertension (DASH) diet, are associated with reduced risk of death in renal disease [8,11]. One of the proposed mechanisms of mediated risk is through higher intake of fruits and vegetables, which are inherently cardio-protective due to their higher levels of dietary fibre, antioxidants, and lower renal acid load [10,11]. In addition, plant-based diets provide an abundant source for a large number of non-nutrient phytochemicals such as carotenoids and polyphenols [12,13].

Polyphenols, present only in plant-based foods, have been associated with reductions in cardiovascular disease and related chronic diseases in large observational studies [14,15,16,17]. Examples of food sources of polyphenols include various berries (hydroxybenzoic & hydroxycinnamic acids), grapes and currants (anthocyanins), onions and kale (flavonols), parsley and celery (flavones), soy products (isoflavones), and fruit juices (flavanones) [18]. The potential mechanisms of action responsible for these cardioprotective effects include their antioxidant and anti-inflammatory properties [19]. Polyphenols may also influence cholesterol levels through modulation of hepatic cholesterol metabolism [20]. Furthermore, animal studies have demonstrated reductions in blood pressure after polyphenol consumption that was associated with endothelium-dependent relaxation and induction of gene expression related to nitric oxide synthase [21]. 

In haemodialysis supplementation studies, key vitamins have demonstrated improvements in (non-polyphenol) antioxidant activity, such as Vitamin C [22] and Vitamin E supplementation [23,24]. While other polyphenol-rich interventions have shown promise to control oxidative stress and ameliorate inflammation in ESKD patients, for example, grape juice powder [25], pomegranate juice [26], turmeric [27], and cocoa flavanols [28].

To date, the effects of polyphenol-rich interventions on CVD risk markers is mixed and no systematic review has specifically evaluated nor pooled the effect of polyphenols on CVD outcomes in dialysis patients. Therefore, the aim of this review was to systematically evaluate the literature from existing randomised controlled trials on polyphenol-rich interventions (food and products) and how it affects CVD markers in haemodialysis populations.

## 2. Methods

### 2.1. Literature Search

This review is written in accordance with the Preferred Reporting Items for Systematic Reviews and Meta-Analyses (PRISMA) guidelines [29]. Relevant studies were identified through a systematic search of the Cochrane Library, MEDLINE (via Scopus), Embase, and CINAHL databases for articles published since journal inception up to the 29 June 2017. Search terms (including mapping to appropriate MeSH terms where appropriate) described major polyphenol classes (‘polyphenol’; ‘phenol’; ‘flavonoid’; ‘flavone’; ‘flavonol*’; ‘isoflavon*’; ‘hydroxycinnamic’; ‘hydroxybenzoic’) and common polyphenol-rich foods (‘Juice’; ‘wine’, ‘tea’; ‘olive’; ‘cacao’; ‘berry’; ‘herb’; ‘spice’; ‘plant’; ‘soy’; ‘flax’; ‘nut’; ‘mint’); in combination with keywords relating to dialysis (‘dialysis’; ‘end-stage renal’; ‘end-stage kidney’; haemodialysis’; ‘peritoneal’; ‘renal failure’; ‘kidney failure’; ESKD’ referring to End Stage Kidney Disease; ‘ESRD’ referring to End Stage Renal Disease). 

Studies were eligible for inclusion if they (1) used a double blind, randomized, placebo-controlled trial study design; (2) had no concurrent intervention; (3) examined the effect of a polyphenol-rich intervention on CVD outcomes (e.g., lipid profile, blood pressure, oxidative stress); and (4) recruited haemodialysis patients only. Other ESKD populations were excluded, in an attempt to keep the study population homogenous. We used the Phenol-Explorer 3.6 database to characterize and inform our decision on known polyphenol-rich interventions [30,31].

### 2.2. Data Extraction

The screening of articles was independently conducted by two review authors (J.K. and W.M.), with disagreements in judgement resolved by consensus or third reviewer (S.M.). Relevant articles titles and abstracts were initially screened. If deemed potentially eligible, studies were selected for full text review. Data was extracted from relevant studies using the following parameters: author/date, study design, sample size, total study period, population characteristics (including age, gender and co-morbidities), intervention characteristics (including type of polyphenol, dose and duration of exposure), length of follow up, and country of origin. For all included studies, mean, standard deviation (SD), standard error or 95% confident intervals (CI) for all pre-specified outcome data that were reported at baseline and follow-up were extracted for analysis if a significant difference was reported. Data was extracted by one reviewer (S.M.) and checked for accuracy by a second reviewer (S.N.).

### 2.3. Assessment of Study and Evidence Quality

Bias assessment was preformed based on the Cochrane Risk of Bias tool [32]. This tool provides criteria for assessing the quality of the included studies. All studies were included in the review regardless of bias rating. A score of ‘high’ indicated a high risk of all bias categories. A score of ‘unclear’ was given when information available was inadequate to correctly comment. A score of ‘low’ indicated low risk of all forms of bias and was the most desirable outcome. 

The certainty in the body of evidence for each CVD outcome category was assessed using the Grading of Recommendations, Assessment, Development and Evaluation (GRADE) assessment tool [33] Certainty in the body of evidence was informed by considering risk of bias, inconsistency, indirectness, imprecision, publication bias, effect size, dose-response and plausible confounding. Based on the pooled or combined data across studies informing these considerations, the certainty in the body of evidence was conserved as very low, low, moderate or high [34]. Determination of the GRADE level of evidence was determined independently by two reviewers (S.M. and J.K.), with disagreements managed by consensus and discussion with a third reviewer (W.M.).

### 2.4. Data Analysis 

The overall treatment effect on primary and secondary outcomes was calculated as the difference between the intervention and comparison groups’ from change scores from baseline to the end of follow-up, or end of intervention values, permitting no significant differences observed at baseline between groups. 

Quantitative analysis was conducted for sufficiently homogeneous and adequately reported outcome measures by pooling data into Review Manager (Version 5.3, The Cochrane Collaboration 2014) for meta-analysis using raw data. The appropriate variance from each individual study was used, either as the SD or calculated from the standard error of the mean (SEM) or 95% CI. Studies that reported on Median and Inter-Quartile-Ranges were assumed to not be normally distributed data and therefore not included in the meta-analysis. Meta-analysis was performed using the DerSimonian and Laird random-effects model [35] 

The *I*^2^ statistic was used to assess the inconsistencies between studies and describe the percentage of variability in effect and data was checked using the fixed-effect model to ensure robustness and susceptibility to potential outliers. Heterogeneity was considered substantial if the *I*^2^ statistic was ≥50%. A statistically significant (*p* < 0.05) result was considered evidence of an effect.

## 3. Results

### 3.1. Study Selection

**Figure 1:**
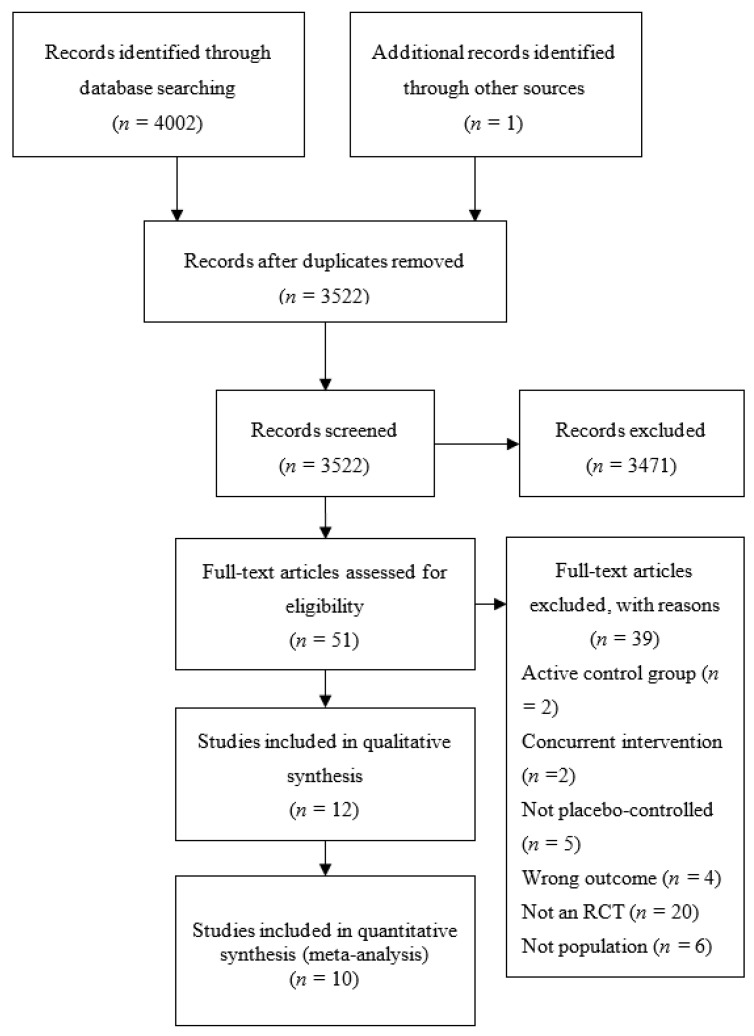
Preferred Reporting Items for Systematic Reviews and Meta-Analyses (PRISMA) study flow diagram describing process of study selection.

As shown in Figure 1, the literature search identified 3521 citations after the removal of duplicates. Initial screening identified 50 papers as potentially relevant for full text review. From this, 39 studies were excluded as they did not meet the inclusion criteria. Hand searching identified 1 additional study for inclusion, leaving 12 total studies included in the review. 

### 3.2. Study Characteristics

Table 1 provides a summary of the study designs of included studies. The total sample size of the included studies was 520 participants, with individual study sample sizes ranging from 27 to 101 participants. All studies used a double-blind, randomized placebo-controlled parallel study design.

### 3.3. Interventions

A variety of interventions were investigated including pomegranate juice (standardized to 0.7 mmol/L polyphenols) [26,36,37], pomegranate extract (standardized to 600–755 mg gallic acid equivalents), cacao (900 mg cocoa-flavanols) [28], turmeric (22.1 mg curcumin) [27,38], grape (500 mg total polyphenols) [25], green tea (455 mg total catechins) [39], and soy (26–54 mg isoflavones) (Table 2) [40,41,42,43]. The duration of the study interventions varied from an acute, one day study [26] to 12 months [36,37]. Interventions were delivered in a juice [26,36,37]; a fortified jelly [25]; drink, cereal or protein bar [40]; powder [28,41,42,43]; or capsules [27,38,44]. Most studies required participants to consume the intervention each day of the study duration with the exception of one acute study where participants consumed pomegranate juice once during the first hour of a haemodialysis session and two long term studies where the intervention was given three or four times per week [26,37,43]. One study reported results as Median and Inter-Quartile-Ranges and were assumed to not be normally distributed data and not included in the meta-analysis [40].

### 3.4. Study Results

#### 3.4.1. Oxidative Stress

Seven studies reported measures of oxidative stress [26,27,28,37,43,44]. A variety of measures were used to assess oxidative stress including advanced oxidation protein products [26,37,44], polymorphonuclear leukocyte priming [26,37], oxidized fibrinogen [37], oxidized LDL-C (Low Density Lipoprotein Cholesterol) [28,43,44] , malondialdehyde (MDA) [27,37], oxygen radical absorbance capacity (ORAC) assay [44], 8-hydroxy-20-deoxyguanosine (8-OHdG) [44], red blood cell catalase [27], glutathione reductase [27], glutathione peroxidase [25,27], and myeloperoxidase [26,37].

Pomegranate juice and extract improved markers of oxidative stress in three studies [26,37,44]. Two studies reported significant reductions in advanced oxidation protein products, polymorphonuclear leukocyte priming, myeloperoxidase [26,37]. One study reported significant reductions in oxidized fibrinogen (*p* = 0.03) and MDA (*p* < 0.001) [37], and another reported a significant interaction effect (group × time) for a measure of HDL-C (High Density Lipoprotein Cholesterol; *p* value not reported) associated paranoxose-1 activity [44].

Soy supplementation reduced oxidized LDL-C in one study (*p* < 0.05) [43], and one study reported turmeric supplementation to improve measures of catalase (*p* = 0.039) and MDA (*p* = 0.040) [27]. No other significant between-group differences in measures of oxidative stress were reported. 

**Figure 2:**
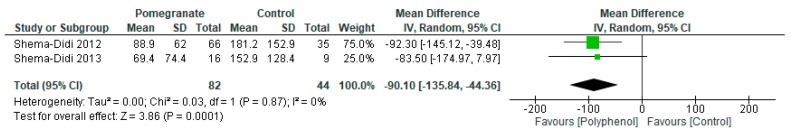
Forest plot of polyphenol-rich interventions on myeloperoxidase.

Meta-analyses reported significant improvements in myeloperoxidase (MD −90.10 (95% CI −135.84, −44.36); *I*^2^ = 0%; *p* = 0.0001; *n* = 2 studies; 1 polyphenol-rich intervention; *n* = 126 participants; Figure 2). There was insufficient data to conduct a meta-analysis on any other measure of oxidative stress due to insufficient numbers of common outcomes reported in the included studies. 

#### 3.4.2. Inflammation 

Eight studies reported measures of inflammation [25,26,28,37,38,40,43,44]. Analyzed markers include C-reactive protein [25,28,38,40,43,44], interleukin-6 [28,37,40,44], tumor necrosis factor alpha (TNF-α) [37,40,43], 8-iso-prostaglandin F2α [43]; and advanced glycation end products [28].

One study reported turmeric to reduce CRP (*p* = 0.012) [38] and one study reported pomegranate juice to reduce IL-6 (*p* < 0.001) and TNF-α (*p* = 0.03) [37] No other significant between-group differences in inflammatory measures were reported. 

Meta-analyses reported no significant difference in CRP (MD 1.31 mg/dL (95% CI −1.11, 3.74); *I*^2^ = 85%; *p* = 0.29; *n* = 5 studies; 5 polyphenol-rich interventions; *n* = 195 participants), IL-6 (MD −0.94 mg/dL (95% CI −2.73, 0.85); *I*^2^ = 88%; *p* = 0.30; *n* = 3 studies; 2 polyphenol-rich interventions; *n* = 128 participants), and AOPP (MD −17.70 mg/dL (95% CI −46.46, 11.06); *I*^2^ = 81%; *p* = 0.23; *n* = 3 studies; 1 polyphenol-rich intervention; *n* = 153 participants). See Supplemental material for forest plots of each non-significant analysis. 

#### 3.4.3. Hemodynamic Measures

Four studies measured at least one of the following haemodynamic measures: flow mediated dilatation [28], augmentation index [44], pulse wave velocity [28,44], pulse pressure [36], intima-media thickness [28,37,44], and measures of blood pressure (i.e., systolic, diastolic, aortic blood pressure) [28,36,44].

Pomegranate extract significantly improved systolic and diastolic blood pressure, and mean arterial pressure (*p* < 0.05 reported for all measures) [44]. Cocoa flavanols significantly improved flow-mediated dilatation (*p* < 0.001) [28]. No other significant between-group differences were reported. 

**Figure 3:**
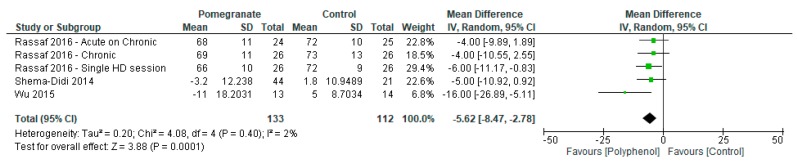
Forest plot of polyphenol-rich interventions on diastolic blood pressure.

Meta-analyses reported significant improvements in diastolic blood pressure (MD −5.62 mmHg (95% CI −8.47, −2.78); *I*^2^ = 2%; *p* = 0.0001; *n* = 4 studies; 2 polyphenol-rich interventions; *n* = 245 participants; Figure 3) but not systolic blood pressure (MD mmHg −10.02 (95% CI −21.39, 1.35); *I*^2^ = 66%; *p* = 0.08; *n* = 4 studies; 2 polyphenol-rich interventions; *n* = 193 participants)

**Figure 4:**
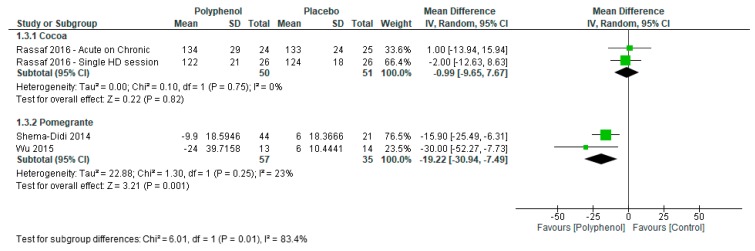
Sensitivity analysis of polyphenol-rich interventions on systolic blood pressure.

**Figure 5:**
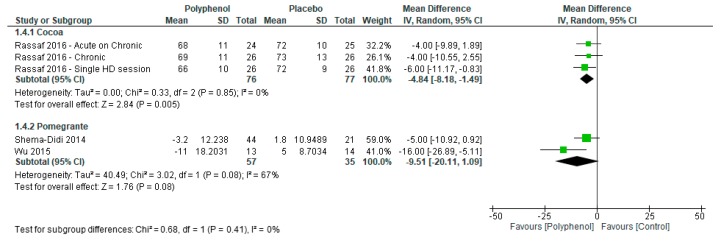
Forest plot of polyphenol-rich interventions on diastolic blood pressure.

Sensitivity analyses were conducted to determine the effect of individual polyphenol-rich interventions on systolic and diastolic blood pressure. Pomegranate (−19.22 (−30.94, −7.49)) had a greater effect on systolic blood pressure compared to cacao (−0.99 (−9.65, 7.67); *p* = 0.01; Figure 4) whereas there was no subgroup difference between pomegranate (−9.51 (−20.11, 1.09)) and soy (−4.84 (−8.18, −1.49)) for diastolic blood pressure (*p* = 0.41; Figure 5).

#### 3.4.4. Lipid Profiles

Four studies reported on changes to cholesterol profile (i.e., total-C, HDL-C, LDL-C and triglycerides) following pomegranate [36,44], and soy supplementation [41,42]. Pomegranate had no significant between-group differences on participant lipid profiles except for one study that reported significant improvements in HDL-C (*p* = 0.03) and triglycerides (*p* = 0.008) for a subset of participants with low HDL-C or high triglycerides, respectively [36]. Soy supplementation improved fasting total cholesterol (*p* < 0.05) in one study and another study reported significant improvements on fasting triglycerides and total cholesterol in a subset of hyperlipidemic participants only (*p* < 0.05) [41,42].

**Figure 6:**
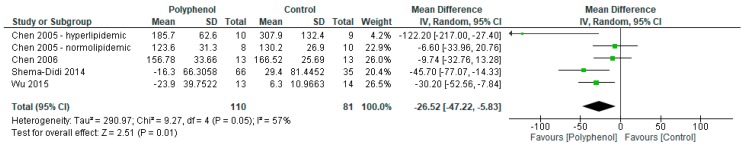
Forest plot of polyphenol-rich interventions on triglyceride levels.

Meta-analyses reported significant improvements in triglycerides (MD −26.52 mg/dL (95% CI −47.22, −5.83); *I*^2^ = 57%; *p* = 0.01; *n* = 4 studies; 2 polyphenol-rich interventions; *n* = 191 participants; Figure 6) but no significant difference in total (MD −11.24 mg/dL (95% CI −24.81, 2.34); *I*^2^ = 76%; *p* = 0.10; *n* = 4 studies; 2 polyphenol-rich interventions; *n* = 191 participants), HDL-C (MD 2.38 mg/dL (95% CI −0.05, 4.82); *I*^2^ = 23%; *p* = 0.06; *n* = 4 studies; 2 polyphenol-rich interventions; *n* = 191 participants), and LDL-C (MD mg/dL −3.31 (95% CI −14.45, 7.84); *I*^2^ = 43%; *p* = 0.13; *n* = 4 studies; 2 polyphenol-rich interventions; *n* = 191 participants). 

**Figure 7:**
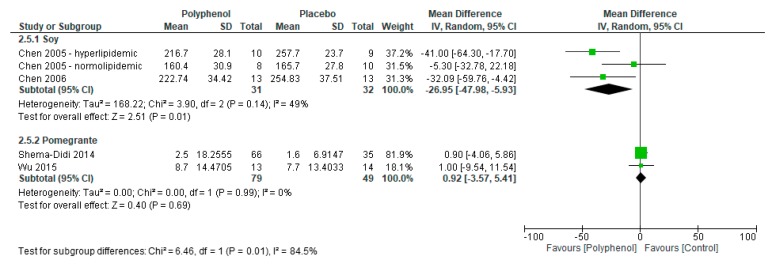
Sensitivity analysis of polyphenol-rich interventions on total cholesterol.

**Figure 8:**
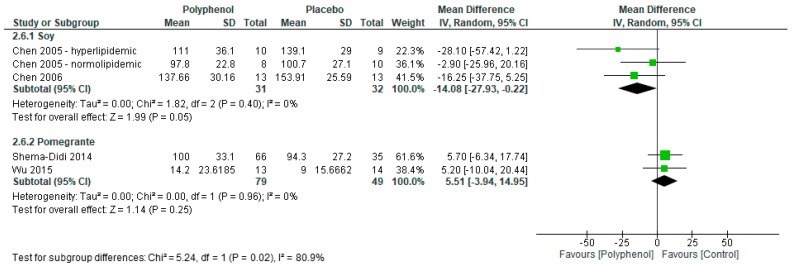
Sensitivity analysis of polyphenol-rich interventions on LDL-C.

Sensitivity analyses were conducted to determine the effect of individual polyphenol-rich interventions on lipid markers. Soy had a greater effect on total cholesterol (−26.95 (−47.98, −5.93) vs. 0.92 (−3.57, 5.41); *p* = 0.01; Figure 7) and LDL-C (−14.08 (−27.93, −0.22) vs. 5.51 (−3.94, 14.95); *p* = 0.02; Figure 8) compared to pomegranate. There were no significant subgroup differences between soy and pomegranate for HDL-C (1.56 (−1.91, 5.03) vs. 3.16 (−2.23, 8.54), respectively; *p* = 0.63) and triglycerides (−20.29 (−54.32, 13.73) vs. −35.42 (−53.63, −17.21), respectively; *p* = 0.44). 

### 3.5. Adverse Events

Eight studies provided data on measured adverse events or irregular biochemistry [26,28,37,38,39,40,45,46]. Most studies did not report any adverse events during the intervention period. Minor gastrointestinal symptoms (e.g., constipation or diarrhea) were reported in one study [25] Severity of adverse events was only reported in one study, which reported one serious adverse event (bleeding) within the intervention group [28]. No study reported a statistical analysis to determine significant differences in adverse event rates between control and interventions arms. Three studies reported changes in potassium levels with all three studies reporting no change after grape powder [25], soy [43], and cacao [28].

### 3.6. Risk of Bias 

**Figure 9:**
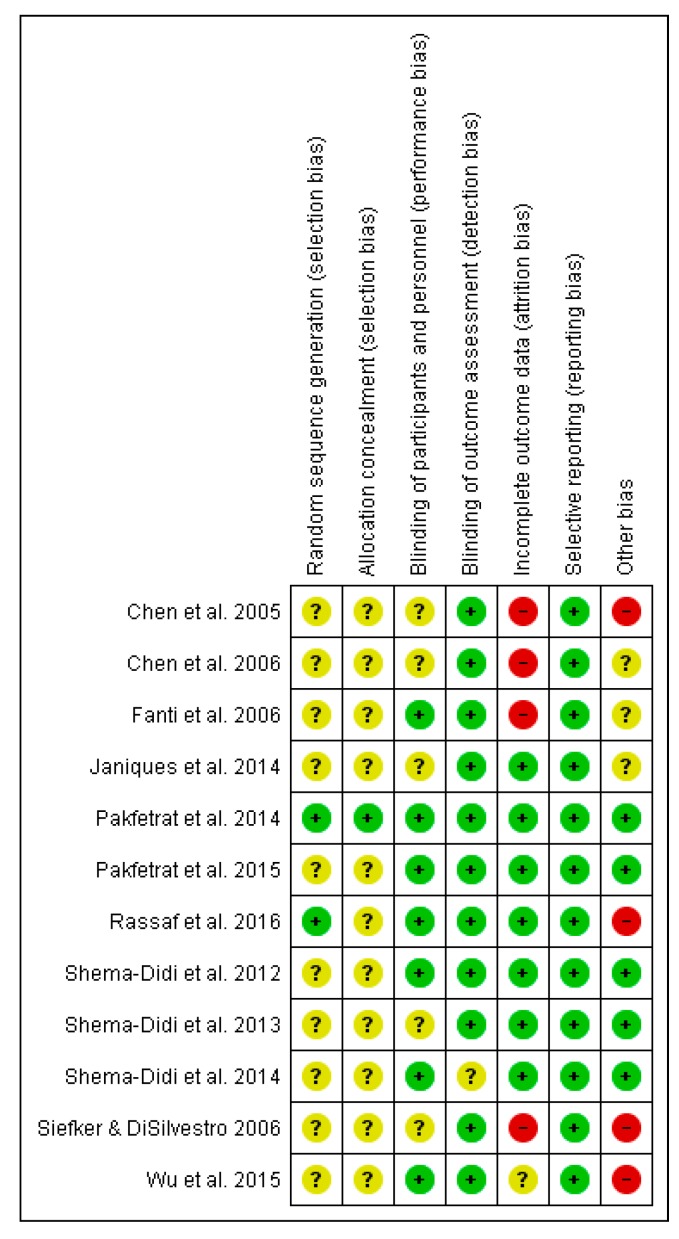
Risk of bias summary across the included studies. Unclear risk of bias: “?”, Low of risk of bias: “+”, High risk of bias: “-“.

Risk of bias was low or unclear for most studies in the following domains: detection (11/12 rated as low) and reporting bias (12/12 rated as low), selection bias (10/13 and 11/12 rated as unclear). Three studies reported high risks of attrition bias and three studies were determined to have other risks of bias such as possible or undeclared conflicts of interest (Figure 9).

### 3.7. Quality of Evidence

Using the GRADE tool, most outcomes were rated at moderate quality (4/12) or very low (5/12) quality with inconsistency and imprecision being the most common reasons for downgrading (Table 3). Of the pooled data with significant findings, there was moderate quality of evidence for the effect on myeloperoxidase (oxidative stress marker); high quality for the effect on diastolic blood pressure, and very low quality for the effect on triglycerides. 

## 4. Discussion

The aim of this systematic literature review was to synthesise results from existing randomized controlled trials to evaluate the effect of polyphenol-rich interventions on cardiovascular markers in haemodialysis patients. The results of individual studies included in this review indicate that polyphenol-rich interventions may improve cardiovascular risk in patients on haemodialysis by improving various markers of inflammation (i.e., CRP, IL-6, TNF-α), lipid profile (i.e., HDL-C and triglycerides), blood pressure, and oxidative stress (i.e., advanced oxidation protein products, polymorphonuclear leukocyte priming, myeloperoxidase, oxidized fibrinogen, catalase, glutathione peroxidase, and MDA); with varying effect sizes and precision across studies. 

Despite individual studies reporting significant improvements, pooled results report no effect for most outcomes excepting myeloperoxidase, diastolic blood pressure and triglycerides. Only myeloperoxidase, a measure of oxidative stress, had a large pooled effect size. In addition, using the GRADE assessment, most outcomes were rated as moderate or very low quality which provides limited confidence that the effect sizes reported in the existing evidence is representative of the true effect. The exception is for diastolic blood pressure, which was rated as high quality.

Individual studies that investigated cacao [28], pomegranate [26,36,37,44], turmeric [27,38], and soy [41,43], reported significant improvements in cardiovascular measures. Sensitivity analyses indicate that some polyphenol-rich interventions may provide greater improvements in cardiovascular markers. However, due to the small number of available studies investigating individual interventions in the haemodialysis population, it is premature to conclude superiority of one polyphenol-rich intervention over another at this time. In addition, while polyphenol-rich interventions reported significant improvements in numerous cardiovascular markers, there was little consistency in reported outcomes between studies that measured the same outcome and/or used the same intervention (e.g., blood pressure in [36,37]). Hence, future studies are required to expand the currently limited evidence base and to address such limitations. 

The low baseline levels of some cardiovascular markers may be a possible explanation for the null findings and/or small effect sizes reported in some included studies and pooled data as it may be unlikely that further reductions are possible. For example, Janiques et al. [25] reported no significant difference in CRP; however, reported baseline levels (range: 2.6–2.6 mg/dL) were in the normal range (<3 mg/dL). In contrast, Paketrat et al. [38] reported significant reductions in CRP in participants that had CRP levels above the normal range (range: 7.0–10.8 mg/dL). This is also supported by the results of Wu et al. [44], Shema-did et al. [36], and Chen et al. [42] that reported greater decreases in blood pressure or cholesterol measures in hypertensive or hyperlipidemic participants, respectively. 

Due to the large number of foods that contain appreciable levels of polyphenols [30], the habitual diet of participants may be a significant influence on study results, if not appropriately controlled for. Few studies included in this review implemented measures to control for this; however, future studies may benefit from implementing methods such as recording habitual diet throughout the study through the use of food diaries and research dietitians as well as educating participants on high polyphenol foods to avoid during the trial duration. 

Few adverse events (predominantly gastrointestinal complains, one significant bleeding event reported [28]) were reported during the included trials which provide preliminary evidence for polyphenol-rich interventions being relatively safe within the haemodialysis population. However, due to the additional dietary restrictions present in this population, close monitoring for adverse events are required with clinical use and future trials are required to further evaluate their safety. In particular, although not reported to significantly affect patients in the included studies, consumption of certain polyphenol-rich food items, such as pomegranate juice, can significantly increase potassium intake beyond what would be typically advised for dialysis patients and therefore, care should be taken with people with history of or at higher risks of hyperkalaemia.

A further consideration for future research is to address the poor bioavailability of specific polyphenols. Resveratrol and curcumin (found in turmeric) [45,46], for example, have been demonstrated in pharmacokinetic studies to have poor bioavailability and a short half-life which has been addressed in several studies by using various methods such as nanoencapsulation, lipid emulsions, and co-administering active compounds that interact with liver enzymes involved in drug metabolism [46,47]. Addressing limitations with bioavailability may provide greater treatment efficacy.

A related research area is to elucidate potential inter-individual differences in polyphenol metabolism as this will inform which patients are likely to benefit from polyphenol-rich interventions. Individual differences in gastrointestinal microbiota appear to significantly influence the metabolism of certain polyphenols [48]. For example, the soy isoflavone, daidzein, is metabolised to (S)-equol in only 25–60% of the population [49]. Metabolism of ellagic acid, found in foods such as pomegranate and berries, can also be affected by microbiota composition, affecting timing, quantity, and types of metabolites excreted [50]. The role of microbiota on polyphenol metabolism in patients with kidney disease may be further complicated due to the possible influence of chronic kidney disease on intestinal microbiota [51,52].

This review includes studies that have used polyphenol-rich interventions. However, food interventions are comprised of several bioactive nutritive (e.g., vitamins and mineral) and non-nutritive compounds (e.g., polyphenols) and therefore, the results of the included studies may have been influenced by these additional compounds. Future trials that use standardized polyphenol extracts are recommended to control for the influence of non-polyphenol compounds.

The findings of this study provide preliminary evidence regarding polyphenol-rich interventions; however, results and conclusions are limited by the heterogeneity of interventions, dosages, and durations as well as variability in the cardiovascular risk of included participants. Although polyphenol-rich interventions have reported benefits in non-ESKD patients, considering the inclusion criteria of this review, generalising results to patients with ESKD or chronic kidney disease who are not receiving dialysis should be avoided until further studies are conducted. Furthermore, studies with large sample sizes are required to sufficiently evaluate the adverse events of polyphenol-rich interventions in this population group.

## 5. Conclusions

This review evaluated the clinical evidence of various polyphenol-rich interventions for patients with ESKD receiving haemodialysis from double-blind placebo-controlled randomized trials. The results of this review provide preliminary support for the use of polyphenol-rich interventions as part of cardiovascular disease prevention and/or management in haemodialysis patients. At this stage, no specific polyphenol-rich intervention appears superior, which is likely due to the small number of available studies, small sample sizes, and lack of control of habitual diet. With this in mind, clinical recommendations are premature until further evidence addresses these limitations. 

## Figures and Tables

**Table 1:** Summary of included studies.

Study	Setting	Study Design	Population
**Soy protein (contains isoflavones)**			
Fanti et al. 2006 [40]	USA Unknown number and type of recruitment sites Data collected: dates not specified	Two-arm parallel, placebo-controlled, double-blind randomised trial Allocation method: random allocation 2:1	ESRD on haemodialysis. Primary disease 44% DM, 36% HTN, 20% other. *n* = 32 participants; *n* = 19 IG; *n* = 13 CG. Attrition: 22% (*n* = 15 IG; *n* = 10 CG) Mean age 61.0 ± 2.9 years; 40% female.
Chen et al. 2005 [42]	Taiwan 1 hemodialysis centre, from a general hospital. Data collected: dates not specified	Two-arm parallel, placebo-controlled, double-blind randomised trial Allocation method: random allocation 1:1	ESRD on haemodialysis. Primary diseases not described. *n* = 42 participants; *n* = 18 IG; *n* = 19 CG (post attrition numbers). Attrition: 12%. Participants divided into normolipidemic (*n* = 10 CG; *n* = 9 IG) and hyperlipidemic (*n* = 9 CG; *n* = 10 IG) Mean age 59–66 ± 9–13 years; 92% female.
Chen et al. 2006 [41]	Taiwan 1 hemodialysis centre, from a general hospital. Data collected: dates not specified	Two-arm parallel, placebo-controlled, double-blind randomised trial Allocation method: random allocation 1:1	ESRD on haemodialysis and all with hypercholesterolaemia. Primary diseases not described. *n* = 29 participants; *n* = 18 IG; *n* = 19 CG (post attrition numbers). Attrition: 11%. Participants divided into normolipidemic (*n* = 13 CG; *n* = 13 IG) and hyperlipidemic (*n* = 9 CG; *n* = 10 IG) Mean age 58–59 ± 11–12 years; 27% female.
Siefker & DiSilvestro 2006 [43]	USA 2 hemodialysis centres, from 2 general hospitals. Data collected: dates not specified	Two-arm parallel, placebo-controlled, double-blind randomised trial Allocation method: random allocation 1:1	ESRD on haemodialysis and all with hypercholesterolaemia. Primary diseases not described. *n* = 20 participants; *n* = 8 IG; *n* = 9 CG (post attrition numbers). Attrition: 15%. Mean age 20 years (range 27–77); 59% female.
**Grape (contains various polyphenols)**			
Janiques et al. 2014 [25]	Brazil 1 nephrology centre Data collected: dates not specified	Two-arm parallel, placebo-controlled, double-blind randomised trial Allocation method: random allocation 1:1	ESRD on haemodialysis. Primary diseases not described. *n* = 34 participants; *n* = 17 IG; *n* = 17 CG. Attrition: 6% (*n* = 16 IG; *n* = 16 CG). Mean age 52.7–53.0 ± 9.8–13.7 years; 44% female.
**Turmeric (contains curcuminoids)**			
Pakfetrat et al. 2014 [38]	Iran 1 haemodialysis centre Data collected: 2011–2012	Two-arm parallel, placebo-controlled, double-blind randomised trial Allocation method: random allocation 1:1	ESRD on haemodialysis, all with uremic pruritus. Primary diseases not described. *n* = 100 participants; *n* = 50 IG; *n* = 50 CG. Attrition: 0%. Mean age 51.0–55.6 ± 14.7–16.6 years; 40% female.
Pakfetrat et al. 2015 ^a^ [27]	Iran 1 haemodialysis centre Data collected: dates not specified	Two-arm parallel, placebo-controlled, double-blind randomised trial Allocation method: random allocation 1:1	ESRD on haemodialysis, all with uremic pruritus. Primary diseases: 35% DM, 33% HTN. *n* = 50 participants; *n* = 50 IG; *n* = 50 CG. Attrition: 4%. Mean age 47–52 ± 15 years; 45% female.
**Cocoa (contains flavanols)**			
Rassaf et al. 2016 [28]	Germany 1 renal centre Data collected: 2012–2013	Two-arm parallel, placebo-controlled, double-blind randomised trial Allocation method: random allocation 1:1	ESRD on haemodialysis, all with uremic pruritus. Primary diseases: 32% HTN, 23% DM, 19% GN, 26% other. *n* = 52 participants; *n* = 26 IG; *n* = 26 CG. Attrition: 6% (*n* = 24 IG; *n* = 25 CG). Mean age 65 ± 13 years; 26% female.
**Pomegranate (Phenolic acids & Flavanoids)**			
Shema-Didi et al. 2012 [37]	Israel 1 dialysis centre in a hospital Data collected: dates not specified.	Two-arm parallel, placebo-controlled, double-blind randomised trial Allocation method: random allocation 2:1	ESRD on haemodialysis. Primary diseases: 64% DM, 60% CVD. *n* = 101 participants; *n* = 66 IG; *n* = 35 CG. Attrition: 34% (*n* = 41 IG; *n* = 26 CG). Mean age 66–68 ± 11–13 years; 45% female.
Shema-Didi et al. 2014 [36]	As per Shema-Didi et al. 2012 [37]	Shema-Didi et al. 2012 [37]	Shema-Didi et al. 2012 [37]
Shema-Didi et al. 2013 [26]	Israel 1 dialysis centre in a hospital Data collected: dates not specified.	Two-arm parallel, placebo-controlled, double-blind randomised trial. Allocation method: random allocation 2:1	ESRD on haemodialysis, receiving an iron transfusion during a single haemodialysis session. Primary diseases: 60% DM, 51% CVD. Others not described. *n* = 27 participants; *n* = 17 IG; *n* = 10 CG. Attrition: N/A Mean age 65–67 ± 11–13 years; 40% female.
Wu et al. 2015 [44]	USA Unknown number of dialysis clinics in two cities Data collected: dates not specified.	Two-arm parallel, placebo-controlled, double-blind randomised trial Allocation method: random allocation 1:1	ESRD on haemodialysis. Primary diseases: 49% HTN, 36% DM. Others not described. *n* = 33 participants; *n* = 16 IG; *n* = 17 CG. Attrition: 19% (*n* = 13 IG; *n* = 14 CG). Mean age 53–56 ± 3 years; 39% female.

CVD, Cardiovascular Disease; CG, control group; DM, Diabetes Mellitus; HTN, hyptertension; IG, intervention group; N/A, Not Applicable; ^a^ It is unclear if the sample and study is the same as that described in Pakfetrat et al. 2014 [38]. The samples appear to be the same; where Pakfetrat et al. [27] excluded some participants after recruitment, which could explain slight differences in sample characteristics. However, Pakfetrat et al. 2015 [27] does not refer to the earlier study at all, and therefore it is not clear.

**Table 2:** Included Study Intervention Details and Results.

Study	Intervention	Results
**Soy protein (contains isoflavones)**		
Fanti et al. 2006 [40]	Intervention: Isoflavone-containing soy-based a protein powder mixed into a drink (54 mg isoflavones), a protein bar (26 mg isoflavones) or cereal product (26 mg isoflavones). Comparator: Isoflavone-free milk-based protein powder mixed into a drink, a protein bar or a cereal product. Energy, protein, CHO, fat, Na and K intake equal to intervention product. Dose: one supplement item daily (powder 3 times per week; bar or cereal 4 times per week). Duration: 8-weeks.	**Pro-inflammatory markers:** 8-weeks post-baseline: *Serum CRP* IG median 9.7 (IQR: 5.2–20.7 mg/L) CG median 17.5 (IQR: 9.1–40.7 mg/L) *p* > 0.05 between groups *Ex-vivo peripheral blood IL-6—unstimulated* IG median 10.3 (IQR: 8.0–14.0 pg/mL) CG median 32.7 (IQR: 14.5–86.4 pg/mL) *p* > 0.05 between groups *Ex-vivo peripheral blood IL-6—simulated with lipopolysaccharide* IG median 3249.0 (IQR: 1288.5–4024.4 pg/mL) CG median 2076.3 (IQR: 524.9–3268.2 pg/mL) *p* > 0.05 between groups *Ex-vivo peripheral blood TNF-α—unstimulated* IG median 7.5 (IQR: 6.6–8.1 pg/mL) CG median 9.0 (IQR: 7.3–17.6 pg/mL) *p* > 0.05 between groups *Ex-vivo peripheral blood TNF-α—simulated with lipopolysaccharide* IG median 790.9 (IQR: 445.4–1120.1 pg/mL) CG median 504.6 (IQR: 257.1–975.3 pg/mL) *p* > 0.05 between groups
Chen et al. 2005 [42]	Intervention: 30 g isolated soy protein (36.3 mg isoflavone content; as reported in Chen et al. 2006 [41]) fortified with calcium, mixed with 200 mL fluid. Comparator: 30 g milk protein, with equivalent amounts of calcium, energy, carbohydrate and fat; mixed with 200 mL fluid. Dose: one supplement daily Duration: 12-weeks.	**Normolipidemic Subjects—Lipid profile:** 12-weeks post-baseline: *Fasting serum TG:* IG μ123.6 ± 31.3 mg/dL CG μ130.2 ± 26.9 mg/dL *p* > 0.05 between groups *Fasting serum total cholesterol:* IG μ106.4 ± 30.9 mg/dL CG μ135.7 ± 27.8 mg/dL *p* > 0.05 between groups *Fasting serum HDL-C:* IG μ36.5 ± 11.2 mmol/L CG μ37.8 ± 6.6 mmol/L *p* > 0.05 between groups *Fasting serum LDL-C:* IG μ97.8 ± 22.8 mmol/L CG μ100.7 ± 27.1 mmol/L *p* > 0.05 between groups **Hyperlipidemic Subjects—Lipid profile:** 12-weeks post-baseline: *Fasting serum TG:* IG μ185.7 ± 62.6 mg/dL; ***p* < 0.05 decreased since baseline** (μ333.2 ± 114.6 mg/dL) CG μ307.9 ± 132.4 mg/dL ***p* < 0.05 between groups** *Fasting serum total cholesterol:* IG μ216.7 ± 28.1 mg/dL; ***p* < 0.05 decreased since baseline** (μ265.7 ± 28.2 mg/dL) CG μ257.7 ± 23.7 mg/dL ***p* < 0.05 between groups** *Fasting serum HDL-C:* IG μ39.1 ± 7.6 mg/dL; ***p* < 0.05 increased since baseline** (μ33.3 ± 10.1 mg/dL) CG μ36.8 ± 5.7 mg/dL *p* > 0.05 between groups *Fasting serum LDL-C:* IG μ111.0 ± 36.1 mg/dL; ***p* < 0.05 decreased since baseline** (μ150.6 ± 28.2 mg/dL) CG μ139.1 ± 29.0 mg/dL *p* > 0.05 between groups
Chen et al. 2006 [41]	Supplement: 30 g isolated soy protein (36.3 mg isoflavone content) fortified with calcium, mixed with 200 mL fluid. Comparator: 30 g milk protein, with equivalent amounts of protein, calcium, energy, carbohydrate and fat; mixed with 200 mL fluid. Dose: one supplement daily Duration: 12-weeks.	**Lipid profile:** 12-weeks post-baseline: *Fasting serum TG:* IG μ1.77 ± 0.38 mmol/L CG μ1.88 ± 0.29 mmol/L *p* > 0.05 between groups *Fasting serum total cholesterol:* IG μ5.76 ± 0.89 mmol/L CG μ6.59 ± 0.97 mmol/L ***p* < 0.05 between groups** *Fasting serum HDL-C:* IG μ1.03 ± 0.19 mmol/L CG μ0.98 ± 0.13 mmol/L *p* > 0.05 between groups *Fasting serum LDL-C:* IG μ3.56 ± 0.78 mmol/L; ***p* < 0.05 decreased since baseline** (μ4.31 ± 1.03 mg/dL) CG μ3.98 ± 0.61 mmol/L *p* > 0.05 between groups
Siefker & DiSilvestro 2006 [43]	Intervention: soy protein powder (52 mg isoflavone content) mixed with fluid, artificial chocolate flavoured. Comparator: whey protein powder mixed with fluid, artificial chocolate flavoured. Dose: one supplement 4 times per week Duration: 4-weeks.	**Oxidative stress markers:** 4-weeks post-baseline: *Oxidized LDL-C:* Data not reported—presented graphically only. ***p* < 0.05 IG decrease since baseline.** **Average change *p* < 0.05 between groups** (IG 31.02% vs. CG 11.12%). **Pro-inflammatory markers:** 4-weeks post-baseline: *TNF-α:* IG μ4.1 ± 0.6 pg/mL CG μ5.7 ± 0.8 pg/mL *p* > 0.05 between groups *Plasma CRP:* IG μ11701 ± 4741 ng/mL CG μ4095 ± 1952 ng/mL *p* > 0.05 between groups *8-iso-prostaglandin F_2α_:* IG μ490.0 ± 185.1 pg/mL CG μ442.2 ± 56.7 pg/mL *p* > 0.05 between groups
**Grape (contains various polyphenols)**		
Janiques et al. 2014 [25]	Intervention: 12 g (500 mg total polyphenols) grape powder mixed into grape jelly. Comparator: grape jelly/placebo. Dose: 1 tablespoon of jelly daily consumed in the afternoon. Duration: 5-weeks.	**Anti-inflammatory markers:** Not clear when measured; assumed to be measured at end of treatment (5-weeks post-baseline): *Plasma GSH-Px:* IG μ42.0 ± 43.3 nmol/min/mL; ***p* < 0.05 increased since baseline** (μ16.5 ± 41.0 nmol/min/mL); *p* < 0.05 higher at baseline than CG (IG μ16.5 ± 41.0 vs. CG μ1.0 ± 3.46 nmol/min/mL) CG μ17.8 ± 50.3 nmol/min/mL *p* > 0.05 between groups **Pro-inflammatory markers:** Not clear when measured; assumed to be measured at end of treatment (5-weeks post-baseline): *Plasma CRP:* IG μ2.7 ± 0.3 mg/dL CG μ2.8 ± 0.2 mg/dL; ***p* < 0.05 increased since baseline** (μ2.6 ± 0.3 mg/dL) *p* > 0.05 between groups
**Turmeric (contains curcuminoids)**		
Pakfetrat et al. 2014 [38]	Intervention: capsule of 500 mg (22.1 mg active ingredient curcumin, a polyphenol) turmeric powder Comparator: capsule of starch/placebo. Dose: 3 capsules per day, one with each main meal. Duration: 8-weeks	**Pro-inflammatory markers:** Not clear when measured; assumed to be measured at end of treatment (8-weeks post-baseline): *High-sensitivity CRP:* IG μ7.0 ± 8.9 mg/L; ***p* < 0.003 decrease since baseline** (μ10.8 ± 9.7 mg/L) CG μ9.6 ± 9.5 mg/L *p* > 0.05 between groups ***p* = 0.012 Mean change different between groups** (IG −μ0.8 ± 2.6 mg/L vs. CG μ0.4 ± 8.7 mg/L)
Pakfetrat et al. 2015 [27]	Intervention: capsule of 500 mg (22.1 mg active ingredient curcumin, a polyphenol) turmeric powder Comparator: capsule of starch/placebo. Dose: 3 capsules per day, one with each main meal. Duration: 8-weeks	**Oxidative stress:** 8-weeks post-baseline: *Red blood cell catalase (CAT):* IG μ127.30 ± 19.55 kilounits/g Hb; ***p* < 0.0001 increase since baseline** (μ107.78 ± 14.28 kilounits/g Hb) CG μ141.14 ± 22.72 kilounits/g Hb; ***p* < 0.0001 increase since baseline** (μ109.11 ± 11.78 kilounits/g Hb) **Ratio of change significantly between groups** (IG: μ0.1 ± 0.2 vs. CG: μ0.3 ± 0.2 kilounits/g Hb; *p* = 0.039) *Red blood cell glutathione reductase (GR):* IG μ35.4 ± 18.7 units/g Hb CG μ37.7 ± 19.8 units/g Hb *p* > 0.05 between groups *Red blood cell glutathione peroxidase:* IG μ58.2 ± 46.3 units/g Hb; ***p* < 0.001 increase since baseline** (μ25.6 ± 26.1 units/g Hb) CG μ56.2 ± 28.2 units/g Hb; ***p* < 0.001 increase since baseline** (μ28.6 ± 22.1 units/g Hb) *p* > 0.05 between groups *Malondialdehyde (MDA):* IG μ6.0 ± 2.4 nmol/mL; ***p* < 0.001 decrease since baseline** (μ8.6 ± 1.4 nmol/mL) CG μ7.5 ± 1.1 nmol/mL; ***p* < 0.001 decrease since baseline** (μ7.5 ± 2.5 nmol/mL) **Ratio of change significantly between groups** (IG: μ0.2 ± 0.2 vs. CG: μ0.1 ± 0.1 nmol/mL; *p* = 0.040)
**Cocoa (contains flavanols)**		
Rassaf et al. 2016 [28]	Intervention: 450 mg cocoa-flavanol containing low-energy fruit-flavoured powder to be mixed as a drink. Comparator: low-energy fruit-flavoured powder to be mixed as a drink, matched for energy, protein, CHO, micronutrients, theobromine and caffeine. Dose: Two sachets (900 mg cocoa-flavanols) daily Duration: 30 days	**Haemodynamics markers:** 30-days post-baseline: *Flow-mediated vasodilation of the brachial artery:* IG μ3.4 ± 0.9%; ***p* < 0.001 increase since baseline** (μ3.9 ± 0.8%) CG μ3.5 ± 0.7% ***p* < 0.001 between groups** *Pulse wave velocity:* IG μ11.0 ± 0.9 m/s CG μ12.0 ± 1.1 m/s *p* = 0.32 between groups *Carotid intima-media thickness:* IG μ0.81 ± 0.02 mm CG μ0.79 ± 0.02 mm *p* = 0.28 between groups **Blood pressure** *Systolic blood pressure:* *Acute* IG μ122 ± 21 mmHg; CG μ124 ± 18 ***p* = 0.03 between groups** *Diastolic blood pressure:* IG μ66 ± 1.0 mmHg; CG μ72 ± 9 ***p* = 0.04 between groups** *Chronic* *Diastolic blood pressure:* IG μ69 ± 11 mmHg; ***p* < 0.004 decrease since baseline** (μ73 ± 12 mmHg) CG μ73 ± 13 ***p* = 0.03 between groups** *Acute on chronic* *Systolic blood pressure:* IG μ134 ± 29 mmHg; CG μ133 ± 24 *p* = 0.60 between groups *Diastolic blood pressure:* IG μ68 ± 11 mmHg; CG μ72 ± 10 *p* = 0.86 between groups **Pro-inflammatory markers:** 30-days post-baseline: *IL-6:* IG μ3.3 ± 0.4 pg/mL CG μ3.3 ± 0.6 pg/mL *p* > 0.05 between groups *CRP:* IG μ0.7 ± 0.2 mg/dL CG μ0.8 ± 0.5 mg/dL *p* = 0.77 between groups *AGE CML:* IG μ3438 ± 310 ng/L CG μ3189 ± 324 ng/L *p* = 0.35 between groups **Oxidative stress markers:** At day 30: *Oxidized LDL-C:* IG μ51.2 ± 3.6 units per L CG μ50.1 ± 1.8 units per L *p* > 0.05 between groups
**Pomegranate (Phenolic acids & Flavonoids)**		
Shema-Didi et al. 2012 [37]	Intervention: Commercial pomegranate juice (0.7 mmol polyphenols/100 mL juice). Comparator: Placebo juice containing artificial pomegranate extract, confirmed no polyphenol content. Dose: 100 mL, three times per week. Duration: 12-months.	**Oxidative stress:** 12-months post-baseline: *CD11b:* IG μ20.3 ± 7.4 mean fluorescence intensity CG μ27.1 ± 11.2 mean fluorescence intensity ***p* = 0.002 between groups** *Serum myeloperoxidase (MPO):* IG μ88.9 ± 62.0 ng/mL CG μ181.2 ± 152.9 ng/mL ***p* = 0.002 between groups** *Serum advanced oxidation protein products (AOPP):* IG μ151.2 ± 39.9 μM CG μ177.2 ± 49.7 μM ***p* = 0.005 between groups** *Serum Oxidised fibrinogen:* IG μ0.86 ± 0.26 nmol carbonyls/mg fibrinogen CG μ1.03 ± 0.34 nmol carbonyls/mg fibrinogen ***p* = 0.03 between groups** *Serum Malondialdehyde (MDA):* IG μ3.8 ± 1.3 μM CG μ6.9 ± 2.6 μM ***p* < 0.001 between groups** **Pro-inflammatory markers:** 12-months post-baseline: *Serum IL-6:* IG μ4.1 ± 2.8 pg/mL CG μ7.5 ± 4.5 pg/mL ***p* < 0.001 between groups** *Serum TNF-α:* IG median 3.9 (IQR: 1.6 pg/mL) CG median 6.7 (IQR: 6.8 pg/mL) ***p* = 0.03 between groups** **Haemodynamics:** 12-months post-baseline: *Common carotid intima-media thickness:* Data not reported, only percent of participants with improvements or declines. Data not reported, only percent of participants with improvements or declines.
Shema-Didi et al. 2014 [36]	Shema-Didi et al. 2012 [37]	**Haemodynamics markers:** 12-months post-baseline: *Systolic blood pressure:* IG μ135.7 ± 21.3 mmHg; ***p* < 0.05 decrease since baseline** (μ145.6 ± 21.9 mmHg) CG μ135.6 ± 27.7 mmHg *p* = 0.96 between groups *Systolic blood pressure >140 mmHg at baseline:* IG μ144.0 ± 16.9 mmHg; ***p* < 0.05 decrease since baseline** (μ157.8 ± 12.9 mmHg) CG μ157.8 ± 10.3 mmHg *p* = 0.12 between groups *Diastolic blood pressure:* IG μ67.7 ± 13.8 mmHg CG μ63.8 ± 20.4 mmHg *p* = 0.38 between groups *Pulse pressure:* IG μ68.0 ± 16.6 ***p* < 0.05 increase since baseline** (μ74.6 ± 19.5 mmHg) CG μ68.8 ± 14.2 mmHg *p* = 0.84 between groups **Lipid profile:** 12-months post-baseline: *LDL-C:* IG μ100.0 ± 33.1 mg/dL CG μ94.3 ± 27.2 mg/dL *p* = 0.39 between groups *Total-C:* IG μ167.3 ± 43. mg/dL CG μ165.1 ± 35.8 mg/dL *p* = 0.79 between groups *HDL-C:* IG μ36.8 ± 10.8 mg/dL; ***p* < 0.05 increase since baseline** (μ33.1 ± 9.5 mg/dL) CG μ34.3 ± 15.4 mg/dL *p* = 0.40 between groups *HDL-C ≤40 mg/dL at baseline:* IG μ33.6 ± 7.5 mg/dL; ***p* < 0.05 increase since baseline** (μ29.7 ± 5.7 mg/dL) CG μ27.6 ± 11.6 mg/dL ***p* = 0.03 between groups** *TG:* IG μ167.3 ± 86.3 mg/dL; ***p* < 0.05 increase since baseline** (μ183.6 ± 101.6 mg/dL) CG μ206.1 ± 109.4 mL/dL ***p* = 0.05 between groups** *TG ≥200 ng/dL at baseline:* IG μ237.4 ± 102.5 mg/dL; ***p* < 0.05 increase since baseline** (μ310.1 ± 87.8 mg/dL) CG μ320.4 ± 56.8 mg/dL ***p* = 0.008 between groups**
Shema-Didi et al. 2013 [26]	Intervention: Commercial pomegranate juice (0.7 mmol polyphenols/100 mL juice). Comparator: Placebo juice containing artificial pomegranate extract, confirmed no polyphenol content. Dose: 100 mL Duration: Once during the first hour of a haemodialysis session.	**Oxidative stress markers:** At the end of the dialysis session: *Serum myeloperoxidase (MPO):* IG μ69.4 ± 74.4 ng/mL CG μ152.9 ± 128.4 ng/mL; ***p* = 0.04 increased since before dialysis session** (μ95.7 ± 68.8 ng/mL) ***p* = 0.04 between groups** *Serum advanced oxidation protein products (AOPP):* IG μ158.1 ± 55.1 μM CG μ217.5 ± 80.8 μM; ***p* = 0.03 increased since before dialysis session** (μ146.0 ± 19.3 μM) ***p* = 0.04 between groups** **Pro-inflammatory markers:** At the end of the dialysis session: *Serum polymorphonuclear leukocytes (PMNLs):* IG μ4.4 ± 1.7 ×10^3^/UL CG μ4.3 ± 2.6 ×10^3^/UL; ***p* = 0.03 decreased since before dialysis session** (μ5.0 ± 2.8 ×10^3^/UL) *p* = 0.96 between groups
Wu et al. 2015 [44]	Intervention: 1000 mg capsule of purified pomegranate polyphenol extract with 600–755 mg gallic acid equivalents. Comparator: Noncaloric placebo capsule not further described. Dose: 1 capsule daily Duration:6-months	**Haemodynamics markers:** 6-months post-baseline: *Systolic blood pressure:* IG μ133 ± 11.5 mmHg; CG μ133 ± 8.2 mmHg ***p* < 0.05 between groups** (improvement in IG compared with CG over time) *Diastolic blood pressure:* IG μ75 ± 4.4 mmHg CG μ74 ± 3.7 mmHg ***p* < 0.05 between groups** (improvement in IG compared with CG over time) *Mean arterial pressure:* IG μ97 ± 5.7 mmHg CG μ93 ± 4.6 mmHg ***p* < 0.05 between groups** (improvement in IG compared with CG over time) *Carotid intima-media thickness:* IG μ0.76 ± 0.04 mm CG μ0.74 ± 0.06 mm *p* > 0.06 between groups *Aortic pressure wave reflection: Augmentation index normalised to HR 75 bpm:* IG μ34.1 ± 5.6% CG μ21.1 ± 3.6% *p* > 0.05 between groups *Aortic pulse wave velocity:* IG μ11.5 ± 1.6 m/s CG μ10.4 ± 1.4 m/s *p* > 0.05 between groups **Oxidative stress markers:** 6-months post-baseline: *Oxygen radical absorbance capacity (ORAC)* IG μ16,586.9 ± 1827.7 μM CG μ17,415.0 ± 1391.3 μM *p* > 0.05 between groups *Advanced oxidation protein products* IG μ144.4 ± 10.8 μM CG μ141.3 ± 13.2 μM *p* > 0.05 between groups *Oxidized LDL-C* IG μ34.1 ± 6.5 U/L CG μ27.8 ± 4.4 U/L *p* > 0.05 between groups *8-hydroxy-2’-deoxyguanosine (8-OHdG):* IG μ33.1 ± 3.0 ng/mL CG μ31.5 ± 2.9 ug/mL *p* > 0.05 between groups **Pro-inflammatory markers:** 6-months post-baseline: *CRP:* IG μ14.4 ± 4.8 mg/L CG μ4.6 ± 1.5 mg/L *p* > 0.05 between groups *IL-6:* IG μ7.6 ± 1.7 pg/mL CG μ7.4 ± 1.4 pg/mL *p* > 0.05 between groups **Lipid profile:** 6-months post-baseline: *Total cholesterol:* IG μ186.0 ± 18.5 mg/dL CG μ157.3 ± 17.1 mg/dL *p* > 0.05 between groups *LDL-C:* IG μ117.5 ± 18.2 mg/dL CG μ92.0 ± 16.8 mg/dL *p* > 0.05 between groups *HDL-C:* IG μ47.6 ± 4.6 mg/dL CG μ82.0 ± 16.8 mg/dL *p* > 0.05 between groups *TG:* IG μ104.3 ± 22.2 mg/dL CG μ91.1 ± 20.5 mg/dL *p* > 0.05 between groups

AGE CML, Advanced Glycation End product carboxymethyl lysine; CG, Control Group; CRP, C-Reactive Protein; GSH-Px, Glutathione peroxidase; Hb, haemoglobin; IL, Interleukin; IG, Intervention Group; HDL-C, High Density Lipoprotein Cholesterol; LDL-C, Low Density Lipoprotein Cholesterol; TNF-α, Tumour Necrosis Factor Alpha; Total-C, Total Cholesterol; TG, Triglycerides.

**Table 3:** Grading of Recommendations, Assessment, Development and Evaluation (GRADE) assessment of polyphenol supplementation compared to placebo for improving cardiovascular risk factors in haemodialysis patients.

Quality Assessment							Number of Patients		Effect	Quality
Number of Studies	Study Design	Risk of Bias	Inconsistency	Indirectness	Imprecision	Other Considerations	Polyphenol	Placebo	Absolute (95% CI)	
Oxidative stress (myeloperoxidase)										
2	Randomised trials	Not serious	Not serious	Not serious	Serious ^a^	Strong effect	82	44	MD **90.1 SD lower** (135.8 lower to 44.4 lower)	MODERATE
Oxidative stress (other markers—not pooled)										
5	Randomised trials	Not serious	serious ^b^	Not serious	Serious ^c^	None	-	-	See comment	MODERATE
Inflammation (CRP)										
5	Randomised trials	Not serious	very serious ^d^	Not serious	Serious ^e^	None	96	99	MD **1.9 mg/dL higher** (2.2 lower to 6.1 higher)	VERY LOW
Inflammation (IL-6)										
2	Randomised trials	Not serious	very serious ^d^	Not serious	Serious ^e^	Both pomegranate studies	79	49	MD **1.6 mg/dL lower** (5.1 lower to 1.96 higher)	VERY LOW
Inflammation (AOPP)										
3	Randomised trials	Not serious	very serious ^d^	Not serious	Serious ^e^	All pomegranate studies	96	57	MD **17.7 mg/dL lower** (46.5 lower to 11.1 higher)	VERY LOW
Diastolic blood pressure										
4	Randomised trials	Not serious	Not serious	Not serious	Not serious	None	133	112	MD **5.6 mmHg lower** (8.47 lower to 2.78 lower)	HIGH
Systolic blood pressure										
4	Randomised trials	Not serious	Serious ^f^	Not serious	Serious ^e^	None	107	86	MD **10 mmHg lower** (21.4 lower to 1.4 higher)	LOW
Hemodynamic measures (other markers—not pooled)										
4	Randomised trials	Not serious	Serious ^g^	Not serious	Serious ^h^	None	-	-	Not pooled. See Table 2	LOW
Lipid profile (TG)										
4	Randomised trials	Not serious	Serious ^f^	Not serious	Very serious ^e^	None	110	81	MD **26.5 mg/dL lower** (47.2 lower to 5.8 lower)	VERY LOW
Lipid profile (Total-C)										
4	Randomised trials	Not serious	Very serious ^d^	Not serious	Very serious ^e^	None	110	81	MD **11.2 mg/dL lower** (24.8 lower to 2.3 higher)	VERY LOW
Lipid profile (HDL-C)										
4	Randomised trials	Not serious	Not serious	Not serious	Serious ^e^	None	110	81	MD **2.4 mg/dL higher** (0.1 lower to 4.8 higher)	MODERATE
Lipid profile (LDL-C)										
4	Randomised trials	Not serious	Not serious	Not serious	Serious ^e^	None	110	81	MD **3.3 mg/dL lower** (14.5 lower to 7.8 higher)	MODERATE

CI: Confidence interval; dL: decilitre; HDL-C, High Density Lipoprotein Cholesterol; LDL-C, Low Density Lipoprotein Cholesterol; MD: Mean difference; Total-C, Total Cholesterol; TG, Triglycerides. ^a^ Two studies were pooled in a meta-analysis for the outcome of myeloperoxidase. The 95%CI for the outcome was substantial (−135 to −44). ^b^ Although most measures of oxidative stress showed improvement from baseline in the intervention group; a number of control groups also showed improvement in this outcome. Therefore, decreasing confidence in the consistency of results for this outcome. ^c^ Although some had narrow measures of variance, other did show substantial imprecision in their measures of variance. ^d^ Heterogeneity was high (>70%). ^e^ The 95%CI was substantial. ^f^ Heterogeneity was substantial (50–70%). ^g^ Studies showed some improvement in mean arterial pressure and flow-mediated dilation; but other studies found no improvement. No improvement was seen in other measures. ^h^ Although individual studies and outcomes did not show significant variance; there is overall a small number of participants for each outcome as well as combined. This decreases confidence in the precision of the effects.
